# *Nigella sativa*: Valuable perspective in the management of chronic diseases

**DOI:** 10.22038/ijbms.2020.37734.8978

**Published:** 2020-06

**Authors:** Raluca Maria Pop, Adrian Pavel Trifa, Ada Popolo, Veronica Sanda Chedea, Claudia Militaru, Ioana Corina Bocsan, Anca Dana Buzoianu

**Affiliations:** 1 Iuliu Hatieganu University of Medicine and Pharmacy, Department of Pharmacology, Toxicology and Clinical Pharmacology, Cluj Napoca, Romania; 2 Iuliu Hatieganu University of Medicine and Pharmacy, Department of Genetics, Cluj Napoca, Romania; 3 University of Salerno, Department of Pharmacy, Fisciano, Salerno, Italy; 4 Research Station for Viticulture and Enology Blaj, Blaj, Romania

**Keywords:** Anti-inflammatory agents, Anti-oxidants, Cardiovascular diseases, Diabetes mellitus, Leukemia, Lymphoma, Multiple myeloma, Nigella sativa

## Abstract

**Objective(s):**

Over the past 20 years, increasing interest in the use of medicinal plants as alternative or adjuvant treatments of several chronic diseases was observed. Accordingly, *Nigella sativa* or black cumin, a medicinal plant rich in bioactive compounds, has been used worldwide for food purposes or in traditional medicines. This paper aims to reveal *N. sativa* potential as adjunct treatment in cardiovascular diseases, diabetes, and hematological malignancies, due to their increasing prevalence and difficult management in everyday life.

**Materials and Methods:**

Databases like PubMed, Web of Science, Science Direct, Scopus, and Google Scholar were used to search the literature data. Keywords like anti-inflammatory effect, anti-oxidant effect, antihypertensive effects, hypolipidemic effects and hematological malignancies were used in combination with *N. sativa.*

**Results:**

Because of its numerous pharmacological actions, but especially for its anti-oxidant and anti-inflammatory properties, *in vivo* and *in vitro* studies demonstrated *N. sativa* positive effect against diabetes, hypertension, and hypercholesterolemia, all of them associated to cardiovascular diseases progression. Also, it was proved to have marked anti-proliferative, cytotoxic, pro-apoptotic, and anti-metastatic effects, in both solid cancers and hematological malignancies.

**Conclusion:**

*N. sativa* used as complementary treatment to classical medications can improve the management of several chronic diseases.

## Introduction

Plant bioactive compounds are receiving continuous attention because of their diverse pharmacological properties. They are considered very useful in the treatment and prevention of several common diseases and conditions like cancer, cardiovascular diseases, diabetes mellitus, obesity, and neurodegenerative diseases (1–3). Because of their strong anti-oxidant, free radical scavenging, anti-inflammatory, and anti-cancer properties, plants represent an important source of novel therapeutic drug discovery and production. 

*Nigella sativa* L. or black cumin is one of these representative medicinal plants, used for centuries for its health benefits. Systematically *N. sativa* belongs to the *Ranunculaceae* family, and is an annual herb of 8–12 inch height, with pinnate and segmented leaves (4). Its flowers are solitary, self-pollinating, and colored in blue and white. The capsule fruits contain numerous trigonal seeds (4). Because of its seeds that have a specific aroma, a bitter and peppery taste (5, 6), this plant was used worldwide since ancient times not only as condiment and spice (4, 7–11) but also as a therapeutic plant to treat different diseases. Thus, in its originating countries in the south and east of the Mediterranean sea to Iran, Pakistan, and India (5), *N. sativa* is one of the most cherished medicinal species (4), being used to prepare dietary supplemental products, functional cosmetics (10, 12), and also for medicinal purposes like treating gastrointestinal disorders, rheumatism, hypertension, diabetes, obesity (13–15), and respiratory ailments (5, 16). 

The health benefits of NSS different extracts and oils are given by the bioactive metabolites’ presence, either as pure molecules or in a mixture (5, 17–20). The studies show that in the seeds of *N. sativa* there are more than 100 chemical compounds, out of which many still have to be characterized (12, 21, 22) (Table 1). 

**Figure1:**
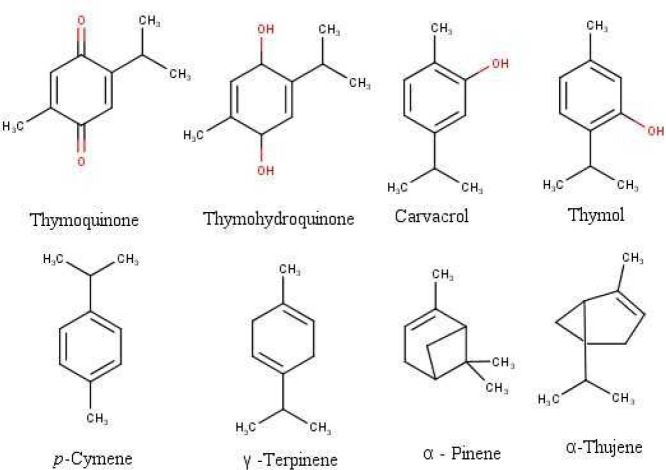
Chemical structure of some principal bioactive compounds in *Nigella sativa*

The most important due to their bioactivity are thymoquinone, nigellone, phytosterols, fatty acids, vitamins, and minerals (23). Thymoquinone (TQ) is the major seed essential oil component and most of the bioactivities have already been attributed to it (22, 24–26). Concerning the *N. sativa* seed oil, its primary components are monoterpenes (87.7%) like p-cymene, carvacrol, α-thujene, terpinene, α-pinene, β-pinene, and its oxygenated derivatives (9.9%) (Figure 1), the rest being sesquiterpenes and derivatives (12, 27). Isoquinoline and indazole alkaloids (7, 28), saponins (29), cycloartenols (30), and flavonoids (11, 30) were also isolated and identified in the seeds.

The *in vivo* and *in vitro* studies showed that among pharmacological properties of NSS, the anti-oxidant and anti-inflammatory activities, anti-asthmatic (31), anti-cancer and anti-diabetic (18, 22, 32–37), anti-hyperlipidemic (38, 39), anti-microbial (40, 41), anti-convulsant (42, 43), and analgesic activities (23, 44, 45) were intensively studied for their therapeutic potential. Thus, these pharmacological properties can be further translated into potent and therapeutically interesting activities on the immune, endocrine, cardiovascular, and respiratory systems as well as into protective effects against different types of induced toxicity (11, 22, 46–49), dermatological (50, 51), and metabolic effects (52–54). Though, a convincing number of studies regarding *N. sativa* health benefits are already available its application in real life disease management is still missing. Thus, the purpose of this review was to provide the most important comprehensive scientific reports that could encourage the clinical use of *N. sativa* especially in cardiology and hematological malignancies. Also, the aim of this review was to analyze the most recent literature data regarding the effects of *N. sativa* on oxidative stress and inflammation in the pathophysiology of chronic disease management. The control of these pathophysiological processes and diseases remains problematic, despite the dedicated effort in this respect. Overall, we aim to show that the use of *N. sativa* finds a great scientific confirmation in preventive medical practice.

## Methods

The artilcles used in the present study were searched using the following databeses: PubMed, Web of Science, Science Direct, Scopus, and Google Scholar. The search was done using the keywords mentioned above. Special foscus was applied to the most recent articles, published between 2010 to 2018, which account for aproximately 65 % out of the total articles used in this review. Articles published between 2000 to 2009 were aproximately 29%. 

***Nigella sativa in inflammation and oxidative stress***

The anti-inflammatory and anti-oxidant effects of *N. sativa* were studied in various acute and chronic models of experimental inflammation and oxidative stress knowing that these two processes are interconnected (55-57). As observed in Table 2, *N. sativa* was used as oil, essential oil, or different types of extracts (water, hydro-alcoholic). 

Also, among bioactive compounds identified in *N. sativa* oil (NSO), TQ was intensively studied (58). Of the vast literature data, the most recent articles, analyzing both inflammation and oxidative stress, were selected to identify how these processes are influencing each other in various illnesses. 

 The anti-inflammatory and anti-oxidant activity of *N. sativa* was observed in an induced model of acute inflammation in rats (59). Within this experimental lipopolysaccharides (LPS) model, the productions of ROS (e.g., nitric oxide (NO)) and of inflammatory mediators (e.g., tumor necrosis factor alpha (TNF-α), interleukins) were stimulated, generating liver and lung inflammation. The groups treated with *N. sativa* [500 mg/kg, intragastric (ig)] and NSO (5 ml/kg, ig) had the FDG (8F-fluoro-deoxy-D- glucose – used to assess the liver and lung inflammation status, with an increased rate in the inflammatory processes) uptakes nearly normal. Regarding the lipid peroxidation, malondialdehyde (MDA) levels decreased in the groups treated with *N. sativa* and NSO. Also, effective anti-oxidant properties were observed in the groups treated with *N. sativa* and NSO, superoxide dismutase (SOD) and catalase (CAT) levels in erythrocyte, lung, and liver being decreased (59).

The effect of NSO on oxidative stress and inflammation was also studied in diabetes-induced rat animal model (60). The oxidative stress parameters (thiobarbituric acid reactive substances-TBARS, NO, Glutathione-GSH levels and Xanthine oxidase -XO, SOD, Glutathione peroxidise-GPx and Glutathione-S-transferase-GST activities) were determined in rat brain tissues. The combination of classical drugs used to treat type2-diabetes mellitus (metformin, glimepiride) with NSO (2.0 ml, orally) had a positive effect on the oxidative stress markers. Therefore, TBARS, NO levels and XO activities, were restored to normal conditions values when compared with the control group. Also, the combination of classical treatment with NSO determined significant elevations of anti-oxidant markers (GSH, GPx, GST, and SOD activities) when compared with diabetic non-treated groups (60). The effect of NSO on central inflammation process (TNF-α, IL-6, IL-1β, and inducible nitric oxide synthase-iNOS) as well as the expression level of nuclear factor kappa-B (NFκ-Bp65) were investigated in both serum and brain tissue. Serum anti-inflammatory levels were significantly lowered in the groups treated with NSO, metformin, glimepiride and their combination as compared with control and diabetic groups. The same trend was observed in the levels of brain inflammatory cytokines. The values were significantly decreased in the rat groups treated with NSO, classical therapy and combination of NSO with classical treatment (60). Thus, NSO, single or in combination with medical treatment suppressed both induced oxidative stress and inflammatory process in type2-diabetes mellitus.

The inflammatory processes, as well as oxidative stress parameters, were also investigated in an experimental autoimmune encephalomyelitis (EAE) animal model in rats (61). The aim was to control the inflammation and demyelination processes in EAE by using *N. sativa* seeds (NSS). As expected, NSS (2.8 g/kg) reduced inflammation and enhanced cerebellum remyelination as confirmed by the histopathological, immunohistochemical, and ultrastructural analysis (61). The anti-inflammatory effect was explained by NSS inhibitory effect on astrocytes expression affecting transforming growth factor beta 1 (TGF-β1) production. The effect of NSS on oxidative stress process was investigated by analyzing the levels of MDA, GSH, GST, NO, and catalase activity in the cerebellum and medulla of rats. A significant decrease of MDA levels was detected in the group fed with NSS 2 weeks prior to EAE induction (cerebellar samples) and in the group fed with NSS after the appearance of first clinical signs (medulla samples). Decreased catalase activity was observed for both cerebellar (groups fed with *N.sativa* after EAE induction) and medulla (groups fed with *N. sativa* both prior and after EAE induction) samples. Regarding GSH levels, the cerebellum samples had a significant increase in both *N. sativa* groups as compared with the EAE group. Significant increases were observed in the medulla samples too, but just for the group treated with *N. sativa* after EAE induction as compared with control levels (61). The promising results obtained in both groups treated with *N. sativa* prior to and after EAE induction suggests that *N. sativa* can be used in prevention and treatment of EAE.

The effect of methanolic extract of *N. sativa* on inflammation and oxidative stress parameters were investigated in clinically endometritic cows (62). The anti-inflammatory effect of *N. sativa* was determined in relation to haptoglobin (Hp) and serum amyloid A (SAA) levels while under oxidative stress through MDA, SOD, and GSH-Px levels (62). The intrauterine infusion of *N. sativa* methanolic extract (100 ml, once a day) exerted anti-inflammatory effects. Thus, the levels of Hp and SAA significantly decreased in the group treated with *N. sativa* as compared with control and ceftiofur hydrochloride groups (62). Oxidative stress induced process within the inflammation model was characterized by significant increases of GSH-Px and SOD levels and by the significant decrease of MDA levels in the groups treated with *N. sativa* and ceftiofur as compared with the control group C. Also, the insignificant differences between *N. sativa* and ceftiofur groups in relation with MDA, SOD, and GSH-Px levels were attributed to the anti-oxidant property of *N. sativa* (62).

The effect of *N. sativa* hydro-alcoholic extract on inflammatory (IL 6 and TNF-a) and oxidative stress markers (MDA, total thiol, and SOD) in a lipopolysaccharide inflammation-induced myocardial fibrosis in rats was also studied (63) . Levels of heart IL-6 and TNF α cytokines were reduced when the highest dose of *N. sativa* extract (400 mg/kg) was administrated, as compared with control groups (63). Oxidative stress parameters were characterized by MDA reduction and total thiol, SOD, and catalase concentrations increase in heart samples. Because of these dose-dependent *N. sativa* changes it was concluded that administration of *N. sativa* improved myocardial fibrosis evolution through alteration of oxidative/anti-oxidative balance.

TQ, the major bioactive compound in *N. sativa* was also studied in different models of inflammation in relation to the oxidative stress processes (16, 64). The response modulation of oxidative stress and inflammatory cytokine profile by TQ was investigated using the collagen-induced arthritis animal model in rats (65). Next, oxidative stress was monitored through myeloperoxidase-MPO, TBARS, lipid peroxidation-LPO, GSH, CAT, SOD, and NO measurement (65). The inflammatory process was followed through inflammatory mediators like IL-1β, IL-6, TNF-α, IL-10, interferon-gamma-IFN-γ, and Prostaglandin E2 - PGE2. They suggested that TQ (5 mg/kg body weight) modulated the antiarthritic effect in joint cartilage through significant suppression of LPO and MPO activity. Decreased NO levels, increased anti-oxidant enzymes activity, the cytokine imbalance, as well the inhibitory effect on the accumulation and activation of polymorphonuclear cells (PMNs) suggested TQ’s antiarthritic effect (65).

The anti-inflammatory molecular mechanism of TQ was studied using complex *in vivo* and *in vitro* experimental settings (66). Within the *in vitro* lipopolysaccharide (LPS) -activated macrophages, and *in vivo* induced hepatitis (LPS/D-galactosamine and induced gastritis (EtOH/HCl) mouse models, TQ anti-inflammatory effect and its target proteins were approached (66). They showed that TQ (25 µM) strongly inhibited NO production, repressed NO synthase, TNF-α, cyclooxygenase (COX−2), IL−6, and IL-1β expression during *in vitro* experiments (66). During *in vivo* experiments, TQ (5 and 25 mg/kg) inhibited NO and PGE2, as well as IL-6 and TNF-α, and the genes responsible for iNOS and COX-2 production (66). They also showed that the inflammatory response in the pancreatic ductal adenocarcinoma was reduced due to suppression of inflammatory gene expression mediated through NF-κB (66). Finally, they proposed that TQ anti-inflammatory effects are probably due to degradation of interleukin-1 receptor-associated kinase 1(IRAK1) and by this the downstream reduction of NF-κB and of activator protein (AP-1) (66).

It can be concluded that *N. sativa* and its derivatives (oil, seeds, extracts, and its active ingredients) exert anti-inflammatory and anti-oxidant effects in the chronic inflammation processes, commonly encountered in many pathological diseases. Generally, the inflammatory process is described in relation with the specific changes in the central or local inflammatory cytokine profile (e.g., IL-6, Il-1β, TNF α, IL-10, INF-γ, and others) while, the oxidative stress process is commonly characterized using parameters like MDA, SOD, CAT, TBARS, NO, GSH, GST, and XO.

 Therefore, according to the literature studies, the use of *N. sativa* in the prevention and treatment of chronic inflammatory disorders represents a very good promise. However, more studies are needed to fully understand how *N. sativa* could be used to control and reduce the inflammation and oxidative stress processes. So far, no clinical trial data are available, thus active research is essential. Since both processes are strongly linked and simultaneously described to initiate and maintain many pathological conditions, it would be very useful that clinical studies would approach the role of *N. sativa* in the chronic disease management from this perspective. 

***Nigella sativa and cardiovascular diseases***

Cardiovascular diseases (CVD’s) are still considered the leading cause of morbidity and mortality in both developed and developing countries. According to the World Health Organization (WHO), every year about 17.1 million people die with cardiovascular problems like heart attack, stroke, and heart failure (67, 68). It is supposed that the occurrence of deaths may increase to 23.6 million in 2030 (WHO 2009). CVD is the leading cause of death worldwide mainly associated with various factors such as elevated total cholesterol serum, raised LDL and increase in LDL oxidation, high platelet aggregation, hypertension, and smoking (69). Besides this, augmented oxidative stress also plays a crucial role in the pathophysiology of CVD such as atherosclerosis, heart failure, hypertension, cardiac hypertrophy, and ischemia-reperfusion (70). Also, oxidative stress and inflammation are responsible for vascular damage common to hypertension, hyperlipidemia, and diabetes. The control of these pathologies is of fundamental importance as they can degenerate into CVDs that are the main cause of death in Western countries.

There are several synthetic therapeutic agents available for the treatment of different cardiovascular disorders at the clinical level, but most of them are facing the problem of unwanted or side effects, inefficacy in a variety of patients, and pharmacokinetic problems. Moreover, the interaction of cardiovascular medication with other drugs also limits the use of certain medications. In this regard, alternative therapies with diverse clinical applications, their effect in multiple patients together with their reasonable safety profile are the target of several research groups around the world. Thus natural healing agents could be considered an ideal approach to these limitations.

**Figure 2:**
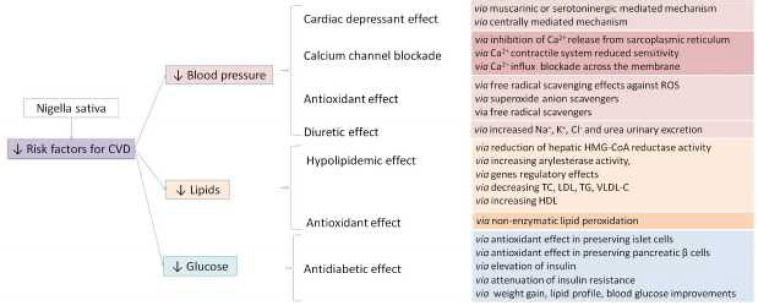
Proposed mechanism of *Nigella sativa* in relation to cardiovascular diseases

Among all natural products used in traditional medicine, *N. sativa* and its constituents showed interesting activity against all cardiovascular risk factors both for their direct pharmacological actions and for its anti-oxidant capacity (Figure 2).

***Antihypertensive effects of Nigella sativa***

It has been reported that the volatile oil of *N. sativa* and TQ, the most pharmacologically active ingredients found in the black seed, decrease both the arterial blood pressure (BP) and heart rate (71)including stroke, coronary artery disease, heart failure, and peripheral vascular disease. The increase in oxidative stress has been associated with the pathogenesis of hypertension. Increase of blood pressure is due to an imbalance between anti-oxidants defence mechanisms and free radical productions. Excessive production of reactive oxygen species reduces nitric oxide bioavailability leading to an endothelial dysfunction and a subsequent increase in total peripheral resistance. Hypertension can cause few symptoms until it reaches the advanced stage and poses serious health problems with lifelong consequences. Hypertensive patients are required to take drugs for life to control the hypertension and prevent complications. Some of these drugs are expensive and may have adverse reactions. Hence, it is timely to examine scientifically, complimentary therapies that are more effective and with minimal undesirable effects. *Nigella sativa* (NS. In a recent systematic review , the results of 11 published studies on humans exploring the BP-lowering effects of *N. sativa* treatment have been reported (72). Meta-analysis showed that *N. sativa* treatment for a period of 4–12 weeks is able to reduce both systolic BP and diastolic BP (72). Furthermore, a subgroup analysis revealed that *N. sativa* powder seems to have more beneficial effects compared with NSO. The exact mechanism involved in the *N. sativa* antihypertensive action is not well established. Many hypotheses have been extrapolated from the results of animal studies and they include a cardiac depressant effect, calcium channel blocking property, and a diuretic effect. The cardiac depressant effect of *N. sativa* seems to be mediated via central mechanisms involving vasomotor center in the medulla and sympathetic outflow to periphery rather than to NO or eicosanoid (71) including stroke, coronary artery disease, heart failure, and peripheral vascular disease. The increase in oxidative stress has been associated with the pathogenesis of hypertension. Increase of blood pressure is due to an imbalance between anti-oxidants defence mechanisms and free radical productions. Excessive production of reactive oxygen species reduces nitric oxide bioavailability leading to an endothelial dysfunction and a subsequent increase in total peripheral resistance. Hypertension can cause few symptoms until it reaches the advanced stage and poses serious health problems with lifelong consequences. Hypertensive patients are required to take drugs for life to control the hypertension and prevent complications. Some of these drugs are expensive and may have adverse reactions. Hence, it is timely to examine scientifically, complimentary therapies that are more effective and with minimal undesirable effects. Nigella sativa (NS. The blockage of calcium channels is mainly due to thymol, one of the active compounds of NS. Thymol reduces Ca^2+^ currents through L-type Ca^2+^ channels, thus inducing vascular smooth muscle cell relaxation. It has also been observed that thymol exerts a negative inotropic action on canine and guinea pig-isolate cardiac preparations (73). Finally, it has been demonstrated that *N. sativa* increases Na^+^, K^+^, and Cl^-^ excretion (74). This diuretic effect enhances the other hypotensive effects described above. 

Alongside these described effects, it is important to underline how the anti-oxidant effect of *N. sativa* is important for limiting hypertension. It is well known that oxidative stress influences the pathogenesis of essential hypertension or the arterial damage produced during essential hypertension (75). In fact, oxidative stress may play a pivotal role in the development of hypertension via the following mechanisms: enhanced sequestration of NO by ROS (76), formation of lipid peroxidation products (77), and depletion of NOS cofactors such as tetrahydrobiopterin (78). Lastly, it may cause functional and structural changes in the vascular wall and blood vessels (79). Among all constituents of NS, TQ seems to be the one mainly involved in this anti-oxidant effect at the base of the anti-hypertensive effect. TQ improves endothelial function by reducing oxidative stress and improving the expression levels of eNOS (17). Furthermore, it has been proven that NSO is able to mediate an anti-hypertensive effect as it reduces the angiotensin-converting enzyme (ACE) activity and increases the heme oxygenase (HO-1) activity. ACE promotes the production of angiotensin II (Ang II), which has a direct vasoconstrictor effect on the vessels but which is also responsible for the production of ROS, which exacerbates vascular damage, while HO-1 reduces the activity of Ang II (75).

***Hypolipidemic effects of Nigella sativa***

Many studies report that *N. sativa* significantly affects the lipid profile by reducing serum cholesterol LDL and triglycerides levels (80) both in animals and in humans. In fact, it has been reported that the petroleum ether extract of *N. sativa* (2 g/kg/day) reduces plasma triglycerides and increases HDL-cholesterol in rats (37) and similar effects have been observed in normal rats treated with NSO. In rabbits fed a cholesterol-rich diet, TQ consumption (3.5 mg/kg, orally) reduces total cholesterol, LDL, and triglycerides and increases HDL-cholesterol concentration (80, 81). A randomized placebo-controlled clinical trial showed that *N. sativa* consumption (2 g/day for 4 weeks) has beneficial effects in the treatment of hypercholesterolemia (82). Another study conducted on hypercholesterolemic patients showed that a dose of 1 g/day of *N. sativa* powder for a period of two months induces a significant decrease in triglyceride and LDL-cholesterol levels, as well as an increase in HDL-cholesterol levels (83). The effects of powdered *N. sativa* (1 g/day) have also been evaluated in menopausal women, one of the high-risk groups for developing dyslipidemia. Results showed a significant decrease in TG, LDL, and total cholesterol levels (84). A large randomized clinical trial evaluated if *N. sativa* supplement (500 mg/day) increases the effect of simvastatin, a widely used hypolipidemic drug. Data from this study showed a significant reduction in cholesterol, TG, and LDL levels in *N. sativa* plus simvastatin-treated patients compared with patients treated with simvastatin alone (85). The mechanisms by which *N. sativa* exerts this hypolipidemic effect are numerous but are all hypothesized based on animal studies. It has been proposed that *N. sativa* reduces the synthesis of cholesterol by hepatocytes and lowers its adsorption from the small intestine (86). It was also proposed that *N. sativa* stimulates cholesterol secretion in the bile (87). Furthermore, anti-oxidant components present in *N. sativa* can prevent non-enzymatic lipid peroxidation, which is a key factor in the atherosclerotic process (88). In addition, the inhibition of the inflammatory mediator’s production plays an important role in the prevention of endothelial dysfunction. Inflammatory cells such as polymorphonuclear leukocytes and vascular endothelial cells are activated in hypercholesterolemia and cause overproduction of free radicals. These free radicals are, in turn, implicated in the initial and development stages of atherosclerosis by oxidation of Ox-LDL and contributed to the inflammatory state of atherosclerosis (89). *N. sativa* reduces the foam cell formation in the blood vessel wall that accelerates the local inflammatory response that eventually leads to atherosclerotic plaque formation (90). Furthermore, it has been reported that TQ improved high cholesterol in the blood and prevented making plaque through decreasing oxidative stress and lipid profiles (91). 

***Anti-diabetic effects of Nigella sativa***

*N. sativa* shows interesting anti-diabetic properties, as reported by several studies. In patients with type 2 diabetes, supplementation of 26.7 mg/kg/day of *N. sativa* seed for 12 weeks resulted in a significant reduction in fasting blood glucose, 2 hr post-prandial blood glucose, glycosylated hemoglobin, and insulin resistance, without affecting renal or hepatic functions (87). A subsequent study, conducted by the same research group, confirmed these favorable effects and showed that they also persist along the one year study period (92). Similar results were obtained in another study in which patients with type 2 diabetes received 30 mg/kg/day of NSO for 3 months in addition to their anti-diabetic drug (93). Studies conducted on animals have allowed highlighting that *N. sativa* inhibits sodium-dependent absorption of D-glucose (54), enhances glucose-induced insulin release from Langherans islets (94), and stimulates glucose uptake in skeletal muscles and adipocytes (95). The common denominator of all these observed effects is the reduction of oxidative stress. In fact, free radicals cause over generation of ROS, which initiates several pathways related to the inflammatory signaling cascades which will lead to inflammation (96) finally causing the apoptosis of pancreatic β cell (97, 98). Thus, *N. sativa* seed, NSO and TQ decrease oxidative stress therefore preserving the pancreatic β cell integrity (80, 99) and increasing peripheral insulin sensitivity (92). Furthermore, *N. sativa* reduces the cardiovascular complications associated with diabetes as it preserves the integrity of the vessel wall as described above (17).

The multiple uses of *N. sativa* in folk medicine encouraged many investigators to isolate its active components in order to evaluate their effects in cardiovascular disorders. The analysis of the data reported in the literature shows that the efficacy of *N. sativa* in these pathologies is mainly ascribed to its anti-oxidant activity. However, some aspects limit the possibility to draw unequivocal conclusions. First aspect refers to the large number of *in vitro* and *in vivo* studies that have been conducted and by contrast to the few in humans. Second aspect concerns the fact that there is not a general formulation for *N. sativa* administration, sometimes being used as powder, other times as an oil, and in many cases only TQ from *N. sativa* was analyzed. The third aspect refers to the different doses used, and to the fact that sometimes these are not well defined. So, long-term human trials are required to better establish the pharmacological activity of *N. sativa* in cardiovascular disorders, diabetes, or to a cluster of metabolic conditions that eventually affects the heart disease management.

***Nigella sativa and hematological malignancies***

Hematological malignancies are cancers broadly divided based on their origin (myeloid and lymphoid) and evolution (acute or chronic). They represent around 10% of all cancers, representing thus an important problem (100)>2000 diagnoses annually, socio-demographically representative of the UK. Their prevalence is expected to increase, as the life expectancy increases and the population gets older. Major types of hematological malignancies include non-Hodgkin lymphoma, multiple myeloma, chronic myeloid leukemia, acute myeloid leukemia, chronic myeloid leukemia, and myelodysplastic syndrome. Although sometimes the hematological malignancies are indolent, most of the patients will need treatment. Treatment-related toxicity can be a major issue in some patients, usually the treatment consisting in prolonged exposure to various combined chemotherapy regimens.

The anti-cancer effects of *N. sativa* and TQ were analyzed *in vitro* and *in vivo*, in relationship with various types of cancers, mostly solid ones. Most of them showed unequivocally anti-proliferative, cytotoxic, pro-apoptotic, and anti-metastatic effects (101). A limited number of studies evaluated the relationship between TQ and hematological malignancies. As these studies were not systematically reviewed, we chose to focus on them in this review, rather than on studies performed on solid cancers.

***TQ effects on acute leukemia cells***

Acute myeloid and acute lymphoblastic leukemias have a poor prognosis, despite the progress made regarding diagnosis and treatment. Acute myeloid leukemia is the most frequent leukemia in adults, its incidence increasing with age. However, in children, acute lymphoblastic leukemia is several times more frequent than acute myeloid leukemia. El-Mahdy *et al.,* (2005) (102) were among the first to provide evidence on how TQ induces apoptosis in leukemic cells. They analyzed the p53-null myeloblastic leukemia HL-60 cell line. They showed that TQ (25, 50 and 100 µM) had a dose- and time-dependent cytotoxic effect on the cells, the IC_50_ (half-maximal inhibitory concentration) being achieved at 23 μM, after 24 hr of TQ exposure. TQ also disrupted the mitochondrial membrane, favored cytochrome c release into the cytoplasm, activated caspases 3, 8, and 9, pro-apoptotic protein Bax up-regulation, and anti-apoptotic protein Bcl-2 down-regulation (102). The effects of TQ (0, 1, 3, 10, 20, 30, 50, 100 µM) on proliferation and apoptosis, using a p53-mutated clone of the human leukemic T-cell line Jurkat were investigated (103). Following the exposure to TQ for 24 hr, both the proliferation and viability of the Jurkat cells were diminished in a dose-dependent manner, the IC50 being 24.2 +/- 0.2 μM. Next, the authors focused on the apoptotic mechanisms triggered by TQ. First, they observed an increasing number of sub-G0/G1 cells (a sign of apoptosis), in parallel with increasing doses of TQ. They went on to demonstrate that TQ stimulates the production of ROS, which destabilizes the mitochondrial membrane. Then, they noted the expression of p73 increment, a protein that regulates cell cycle progression. This was accompanied by a decrease in the expression of UHRF1, HDAC1, and DNMT1 proteins (103)by focusing on the anti-apoptotic and epigenetic integrator UHRF1 which is essential for cell cycle progression. For this purpose, we analyzed the effects of a known anti-neoplastic drug, thymoquinone (TQ. UHRF1 has been shown to have anti-apoptotic properties (104). Thus, its down-regulation would favor apoptosis. In fact, UHRF1 gained considerable interest during recent years. Since it is expressed in most of the cancers, it has the potential to become a “universal” cancer biomarker (104). The same research team that described the relationship between TQ, and p73 and UHRF1 deregulation demonstrated later that this process was preceded by a down-regulation of PDE1A, a cyclic nucleotide phosphodiesterase (105)the active principle of Nigella sativa black seeds, has anti-proliferative properties on numerous cancer cell types. Others and we have previously reported that TQ acts as agent that triggers cell cycle arrest and apoptosis through either a p53- or p73-dependent pathway. However, the immediate targets recruited upon TQ-induced cytotoxicity have not yet been clearly identified. We therefore asked whether cyclic nucleotide phosphodiesterases (PDEs. Among the proteins down-regulated by TQ in Jurkat cells, DNMT1 was mentioned too (103)by focusing on the anti-apoptotic and epigenetic integrator UHRF1 which is essential for cell cycle progression. For this purpose, we analyzed the effects of a known anti-neoplastic drug, thymoquinone (TQ. The effects exerted by TQ on DNMT1 in acute myeloid leukemia cells were demonstrated using *in vitro*, *ex vivo* and *in vivo* models - Kasumi-1, MV4-11, THP-1, and ML- 1 cell lines, primary cells from leukemia patients (with 1, 10, 30, and 300 nM, 1, 3, 10, 30, and 100 μM of TQ) and a murine leukemia model (with 0, 15, and 30 mg/kg TQ, intravenous injection) (106). It was observed that TQ not only binds directly to the catalytic domain of DNMT1, but also diminishes DNMT1 expression. Thus, TQ produces DNA hypomethylation, promoting apoptosis in the leukemic cells. In the light of these findings, TQ could become a DNA hypomethylating adjuvant agent used in the therapy of acute myeloid leukemia, along with the already approved DNA hypomethylating drugs, namely azacitidine and decitabine (106).

The effects of TQ on acute lymphocyte leukemic cell line (CEMss) were also investigated (107). TQ (0, 5, 10, and 20 μg/ml), had cytotoxic effects, with an IC50 value of 1.5±0.04 μg/ml, after 24 hr of TQ exposure. TQ also increased ROS production and favored apoptosis, in a dose- and time-dependent manner, with increased activities for caspases 3, 8, and 9, decreased expression of Bcl-2, and increased expression of Bax (107). The same research team expanded their work on WEHI-3 cell line, which has features of myelomonocytic leukemia. They analyzed the relationship between TQ and this cell line. They also injected WEHI-3 cells to BALB/c mice, in order to assess the *in vivo* effects of TQ. TQ had cytotoxic and pro-apoptotic effects on WEHI-3 cells, in a dose-dependent manner, the IC50 being 2.0±0.04 µg/ml, after 24 hr of exposure. TQ also reduced the size of spleen and liver of BALB/c mice injected with WEHI-3 cells (108).

The propensity of TQ to trigger apoptosis by generating ROS was demonstrated by using TQ (1, 5, 10, 40, or 100 µM) on several leukemic T-cell lines, HTLV-1 positive (MT-2 and HuT-102), and HTLV-1 negative (CEM and Jurkat) (109). TQ were toxic to HTLV-1 negative cells and also to HTLV-2 positive cells, although to a lesser extent. After 48 hr of TQ exposure, IC50 had the following values: 8 μM (CEM), 28 μM (Jurkat), 35 μM (MT-2), and 85 μM (HuT-102). More HTLV-1 negative cells than HTLV1 positive accumulated in sub-G1 phase, suggesting that the malignant HTLV-1 negative cells are more prone to TQ-induced apoptosis. This was confirmed by several other data generated by the study. While TQ induced modifications in both HLTV-1 positive and negative cells, the effects were stronger in HLTV1-positive cells, which displayed: more mitochondrial membrane potential disruption and release of cytochrome c, more activation of caspases 3 and 9, more production of ROS, and a greater depletion of intracellular glutathione. These effects were not seen in normal PMBC (peripheral mononuclear blood cells), an observation that suggests a selective action of the TQ on the malignant lymphocytes (109). Other two recent studies revealed similar findings regarding the effects of TQ on leukemic T cell lines, namely decreased cell viability and induction of apoptosis (110, 111)a natural compound isolated fromNigella sativa, induces growth inhibition and apoptosis in several cancer cell lines. The aim of the present study was to investigate the effect of TQ alone and in combination with doxorubicine on the proliferation inhibition and apoptosis induction of TQ in a lymphoblastic leukemia cell line. Jurkat cell line was cultured in standard condition and with concentrations of TQ (0-30 μm. The involvement of the TGF family, TQ producing up-regulation of TGFβ1, and down-regulation of TGFα (111)there is no effective treatment for ATL. Thymoquinone has been reported to have anti-cancer properties. Objective The aim of this study is to investigatthe effects of TQ on proliferation, apoptosis induction and the underlying mechanism of action in both HTLV-1 positive (C91-PL and HuT-102 was also shown.

***TQ effects on lymphoma cells***

 The possible inhibitory role of TQ on lymphoma cells was analyzed through the effects of TQ on several pleural effusion lymphoma (PEL) cell lines such as BC-1, BC-3, BCBL-1, and HBL-6 (112). PEL is a rare type of lymphoma, appearing mostly in HIV-infected patients. It is associated always with human herpes virus 8. After 24 hr exposure of the PEL cell lines to increasing TQ concentrations (10, 25, and 50 μM), the growth of all PEL cell lines was inhibited by TQ in a dose-dependent manner. Moreover, TQ was non-toxic to non-malignant cells, namely peripheral mononuclear blood cells obtained from healthy individuals. TQ also induced in PEL cell lines several apoptotic mechanisms: inhibition of the PKB/AKT signaling pathway, Bax up-regulation and Bcl-2 down-regulation, mitochondrial membrane disruption and cytochrome c release, caspases 3 and 9 activation, and PARP cleavage. As pre-treatment with N-acetyl-cysteine (a ROS scavenger) abrogates most of these phenomena, it is clear that the apoptosis triggered by TQ greatly depends on ROS produced (112). 

NFkB activation is the hallmark of activated B-cell lymphoma (ABC), one of the main types of diffuse large B-cell lymphoma (DLBCL), which in turn represents the most frequent type of non-Hodgkin lymphoma. The anti-apoptotic role of the NFkB signaling pathway in ABC was investigated based on TQ previously described pro-apoptotic properties (113). Thus, two ABC cell lines, namely HBL-1 and RIVA, were exposed to TQ using concentrations of 5 and 10 μM. TQ shut down the phosphorylation of p65 (a subunit of NFkB). On the other hand, the down-regulation of the p65 targets such as Bcl-2, Bcl-xL, or XIAP favored mytochondrial-induced apoptosis. All these consequences depended on the TQ dose used. TQ produced the release of ROS in ABC cells, mostly superoxide anion and hydrogen peroxide. Pre-exposure of the ABC cells to N-acetyl-cysteine inhibited the release of reactive oxygen species (113). 

Epstein-Barr virus, while asymptomatic in most of the individuals infected, is able to induce several types of lymphoid and epithelial neoplasms, such as Burkitt lymphoma, diffuse large B-cell lymphoma, Hodgkin lymphoma, or nasopharyngeal carcinoma. The relationship between TQ and EBV infected B lymphocytes was investigated (114). Several types of cells: EBV transformed B-cell lines (lymphoblastoid cell lines), an EBV-positive cell line (Raji), two EBV-negative cell lines (DG-75 and BL41), normal PBMC, and periodontal ligament fibroblasts were exposed to TQ (0.4-200 µmol/l) (114). TQ inhibited the growth of both EBV-positive and EBV-negative cell lines. After 24 hr of TQ exposure, the greatest inhibitory effect was seen in the case of lymphoblastoid cell lines (minimum IC50 was 0.99 +/- 0.02 μmol/l), followed by the Raji (EBV-positive) cell line (IC50 was 11 +/- 2.1 μmol/l), followed by the two EBV-negative cell lines (DG-75 and BL41) (IC50 were 20 +/- 2.1 and 16.24 +/- 0.31 μmol/l, respectively). TQ had the lowest toxicity against the normal fibroblasts from the periodontal ligament (IC50 was 85 +/- 6.5 μmol/l). The study revealed also other interesting findings. For instance, TQ induced apoptosis in lymphoblastoid cells, as demonstrated by TUNEL and caspase-3 assays. This effect was dose- and time-dependent. Moreover, TQ also inhibited the expression of several EBV genes, namely EBNA1, EBNA2, and LMP1, the effect being the strongest in the case of EBNA2 (114).

***TQ effects on multiple myeloma cells***

Multiple myeloma (MM) is a mature B-cell lymphoid neoplasm, arising from plasma cells. Although progress has been made regarding the treatment options, it remains a disease with a poor prognosis. Signal transducer and activator of transcription-3 (STAT-3) activation signaling is common to several cancers, including MM. The effects of TQ on STAT-3 signalling, using U266 and RPMI 8226 MM cell lines were analysed (115). TQ had various, multiple effects on MM cells. Firstly, TQ markedly inhibited the constitutive phosphorylation of STAT-3, this effect being dose - and time-dependent. TQ displayed the greatest inhibition at around 15 μM, after around 4 hr of exposure. TQ inhibited not only the constitutive STAT-3 phosphorylation, but also that IL-6-dependent, a cytokine that is crucial in the pathogenesis of MM. The level of several proteins regulated by STAT-3, like cyclin D1, Bcl-2, Bcl-xL, survivin, Mcl-1, and VEGF significantly decreased upon TQ exposure, the effect being the greatest after 36-48 hr (115). Regarding the effects on cell cycle, TQ forced the MM cells to distribute in the pre-G1 phase, a phenomenon indicating apoptosis. In fact, it seems that STAT-3 suppression by TQ promotes apoptosis by activation of caspase-3, which in turn cleaves the PARP protein. 

The effects of TQ on MDN and XG2-MM cell lines was also investigated (116). In general, they observed similar effects of TQ on MM cells, as those reported by Li *et al.* (2010). TQ had dose- and time-dependent cytotoxic effects on both cell lines, the IC50 being 10 μM (MDN cell line) and 8.5 μM (XG2 cell line), respectively. The effect was at peak intensity after 12 hr of TQ exposure. In both MDN and XG2 cells, TQ also inhibited the IL-6-dependent growth. In both MDN and XG2 cell lines, TQ markedly decreased the level of CXCL-12-dependent actin polimerization, a process essential for the dynamics of multiple myeloma cells. Finally, TQ decreased the level of STAT-3 phosphorylation, but not of STAT-5. The level of several proteins regulated by STAT-3 activation, such as Bcl-2 and Bcl-XL was also diminished (116)the major active component of the medicinal herb Nigella sativa Linn., has been described as a chemopreventive and chemotherapeutic compound. METHODS In this study, we investigated the effect of TQ on survival, actin cytoskeletal reorganization, proliferation and signal transduction in multiple myeloma (MM. These observations also parallel the data reported by Li *et al.* (2010).

***TQ as a synergic therapeutic agent in the treatment of hematological malignancies and other cancers***

 The combined effects of TQ and doxorubicin were investigated, and it was found that the two had a synergistic effect (110). This is not surprising, taking into account that the synergism between TQ and various chemotherapeutic agents has been described previously. For instance, Effenberger-Neidnicht and Schobert (2011) analyzed the combined effects of TQ and doxorubicin on several cell lines originating from various human cancers. A synergistic effect between TQ and doxorubicin was noted in the case of HL-60 leukemia cell line and multidrug resistant MCF7/TOPO (topotecan resistant) breast carcinoma cell line (117). A synergism between TQ and gemcitabine and oxaliplatin in pancreatic cancer cells was also reported (118). Importantly, pre-exposure to TQ favored chemosensitization to gemcitabine and oxaliplatin, rather than their co-administration (118). The synergism between TQ and cisplatin in both non-small cell and small cell lung cancer cell lines was described (119). They replicated these *in vitro* data in an *in vivo* mouse xenograft model (119). Next, the synergism between TQ and topotecan, a topoisomerase I inhibitor, in acute myelogenous leukemia cell lines was reported (120). Again, it seems that pre-exposure to TQ before topotecan exposure has more cytotoxic effects than their co-administration (120). TQ also potentiates thalidomide and bortezomib in inducing apoptosis in MM cells (115). The synergism between TQ and bortezomib both *in vitro*, on various MM cell lines, and *in vivo*, on a xenograft mouse model were also investigated (121). Also, the synergistic effect between TQ and temozolomide (TMZ) was reported (122). A higher decrease was observed in brain cancer cell (human glioblastoma cell line (U87MG)) viability when these drugs were used in combination (20 µM of TMZ and 50 µM of TQ) rather than when they were used alone (122). It was suggested that autophagy blockage at the transcriptional level by TQ reduced the resistance mechanism of these cells to TMZ. 

Also, this synergism can be translated in real life by TQ potential to reduce the side effects in different therapies. In this sense, some studies have already revealed promising results. For instance, cardiotoxicity might appear following treatment with anthracyclines, such as doxorubicin. Accordingly, it was demonstrated that TQ could lower the risk of doxorubicin-induced cardiotoxicity, without compromising its anti-cancer properties (123-125). 

Although the number of studies performed on the relationship between *N. sativa* and hematological malignancies is limited, they all demonstrated the anti-proliferative, cytotoxic, and pro-apoptotic effects of its bioactive compound, namely the TQ. However, we should take into consideration that these studies mainly used *in vitro* models. These results should be further explored in *in vivo* models, and then in clinical trials. These clinical trials should probably combine TQ and various chemotherapeutic agents used in the treatment of the hematological malignancies. We could identify several reasons for this approach: first, there is evidence of a synergism between these medicines and TQ, at least *in vitro*. Second, TQ could have the potential to reduce the toxicity associated with various chemotherapeutic agents. Third, the evaluation of TQ alone, as single agent in the treatment of hematological malignancies, would not be acceptable right now, as data regarding TQ action *in vivo* are lacking.

**Table 1:** General composition of *Nigella sativa*

Compound class	Compound name	Content range	Source	Reference
Sterols	% total sterols			
	Cholesterol	0.70-1.28	s	(5, 8, 126–128)
		0.48-1.01	o	
	Campesterol	9.88-13.76	s	(5, 8, 126–128)
		2.28-13.1	o	
	Campestanol	0.54-0.56	s	(5, 8, 126–128)
	Stigmasterol	10.52-20.92	s	(5, 8, 126–128)
		4.31-18.22	o	
	β-sitosterol	44.53-53.95	s	(5, 8, 126–128)
		13.24-53	o	
	-sitosterol	0.59	o	(5, 8, 126–128)
	Sitostanol	2.29-2.38	s	(5, 8, 126–128)
	Δ7-Stigmasterol	1.60-2.22	s	(5, 8, 126–128)
		0.6	o	
	Δ 7-Avenasterol	1.11-2.24	s	(5, 8, 126–128)
		2.1	o	
	Δ 5- Avenasterol	2.1	o	(5, 8, 126–128)
Tocopherols	mg/kg			
	α-Tocopherol	0.8-1.3	s	(129)
	β-Tocopherol	0.5-0.9	s	(129)
	-Tocopherol	1.2-12.1	s	(129)
	β-Tocotrienol	8.2-12.1	s	(129)
Fatty acids	%			
	Lauric acid (C12:0)	0.6	o	(130–132)
	Myristic (C14:0)	0.14-1	s	(5, 128, 133, 134)
		0.15-1	o	
	Palmitic (C16:0)	8.92-10.5	s	(5, 128, 133, 134)
		11.17-13.1	o	
	Palmitoleic (C16:1)	0.18	s	(5, 128, 133, 134)
		0.19-0.2	o	
	Margaric acid (C17:0)	0.061	o	(5, 128, 133, 134)
	Heptadesenoic (C17:1)	0.054	o	(5, 128, 133, 134)
	Stearic (C18:0)	2.04-2.44	s	(5, 128, 133, 134)
		2.22-3.4	o	
	Oleic (C18:1)	9.42-16.23	s	(5, 128, 133, 134)
		22.94-24.64	o	
	Linoleic (C18:2)	63.71-68.07	s	(5, 128, 133, 134)
		55.6-58.5	o	
	Linolenic (C18:3)	0.44-2.16	s	(5, 128, 133, 134)
		2.23-0.4	o	
	Arachidic (C20:0)	0.13	s	(5, 128, 133, 134)
		0.2-0.5	o	
	Eicosenoic (C20:1)	0.27	s	(5, 128, 133, 134)
		0.31	o	
	Eicosedienoic acid (C20:2)	0.33	s	(5, 128, 133, 134)
		2.55	o	
	Behenic (C22:0)	2.89	s	(5, 128, 133, 134)
		0.039	o	
	Docosenoic (C22:1)	0.047	o	(5, 128, 133, 134)
	Lignoceric acid (C24:0)	1.04	s	(5, 128, 133, 134)
Monoterpenic hydrocarbons	mg/100gDW			
	α-Thujene	4.38-109.4	e.o.	(133, 135–137)
	α -Pinene	2.19-27.65	e.o.	(133, 135–137)
	Sabinene	0.39-3.46	e.o.	(133, 135–137)
	β-Pinene	1.1-43.78	e.o.	(133, 135–137)
	Myrcene	0.21	e.o.	(133, 135–137)
	α -Terpinene	0.55-1.15	e.o.	(133, 135–137)
	p-Cymene	11.3-374.4	e.o.	(133, 135–137)
	Limonene	0.31-13.82	e.o.	(133, 135–137)
	-Terpinene	5.21-12.67	e.o.	(133, 135–137)
Monoterpene ester	Bornyl acetate	0.04-1.15	e.o.	(133, 135–137)
Monoterpenoid ketones	Carvone	0.02	e.o.	(133, 135–137)
	p-Cymen-9-ol	12.67	e.o.	(133, 135–137)
	Eugenol	4.61	e.o.	(133, 135–137)
	Thymoquinone	3.52-413.57	e.o.	(133, 135–137)
	Thymohydroquinone	6.35-7.3	e.o.	(133, 135–137)
Monoterpenoid alcohols	Carvacrol	2.61-12.67	e.o.	(133, 135–137)
	4-Terpineol	0.2-9.22	e.o.	(133, 135–137)
Terpenic phenols	Thymol	1.67	e.o.	(133, 135–137)
Sesquiterpene	α-Longipinene	0.34-4.61	e.o.	(133, 135–137)
	Longifolene	1.71	e.o.	(133, 135–137)
	Z-γ-Bisabolene	2.30	e.o.	(133, 135–137)
	(E)-Caryophyllene	0.02-4.61	e.o.	(133, 135–137)
Polyphenols	mg/kg			
	Catechin	56.56-124.6	o	(128)
	Epicatechin	39.67-98.1	o	(128, 138)
		1.28	sh	
		0.64	r	
	Catechin hydrated	7.26	sh	(128, 138)
		3.4	r	
	Rutin	14 -117.7	o	(128, 138)
	Dihydro quercitin	4.05-31.45	o	(128, 138)
	Naringine	2.16-58.68	o	(128, 138)
	Quercimeritrin	5.89-39.89	o	(128, 138)
	Quercetin	5.03-35.67	o	(128, 138)
		2.56	sh	
		2.61	r	
	Apigenin	6.83	sh	(128, 138)
		1.77	r	
	Amentoflavone	2.91	sh	(128, 138)
	Flavone	3.4	sh	(128, 138)
		0.54	r	
	Callistephin	15.99-33.6	o	(128, 138)
	Gallic acid	27.86	sh	(128, 138)
		30.59	r	
	p-Dihydroxybenzoic acid	1.73	r	(138)
	Chlorogenic acid	1.51	sh	(138)
		0.36	r	
	Vanillic acid	143.2	sh	(138)
		89.94	r	
	trans-2-Hydroxycinnamic acid	1.25	sh	(138)
		2.58	r	
	trans-Cinnamic acid	15.47	sh	(138)
		0.98	r	
Macro elements	mg/kg DW			
	Potassium (K)	708-7561	s	(129, 130)
	Magnesium (Mg)	80-1878	s	(129, 130)
	Calcium (Ca)	160-5089	s	(129, 130)
	Zinc (Zn)	2.5-48.89	s	(129, 130)
	Manganese (Mn)	1.5-22.73	s	(129, 130)
	Copper (Cu)	0.9-16.03	s	(129, 130)
	Iron (Fe)	8.65-108.1	s	(129, 130)
	Phosphorus (P)	48.9-6197	s	(129, 130)
Micro elements	mg/kg DW			
	Boron (B)	19.42-23.6	s	(129, 130)
	Chromium (Cr)	0.291-0.65	s	(129, 130)
	Copper (Cu)	15.07-16.3	s	(129)
	Molybdenum (Mo)	0.221-0.40	s	(129)
	Nickel (Ni)	3.49-5.18	s	(129)

S: seeds, o: oil, EO: essential oil, sh: shoots, r: roots

**Table 2:** The anti-inflammatory effects of *Nigella sativa* and TQ, its main constituent

Type	Substance	Administrated dose	Experimental model	Anti-inflammatory effects	Observation	Reference
*In vitro*	Thymoquinone (TQ)		Calcium ionophore stimulated peritoneal leukocytes (rats)	COX and 5-LO pathways of arachidonic acid metabolism were inhibited. Thromboxane, leukotrienes B4, and C4 production was down-regulated.	The effect was dose-dependent. TQ had a higher effect than NSO.	(139)
	*Nigella sativa* oil (NSO)					
	NSO	12.5–50 mg/m	Calcium- or ionophore-stimulated neutrophils		The effect was dose-dependent possibly through the antioxidative action.	(140)
	TQ	0.01 and 6.25 μg				
	Nige llone	6.25 and 50 μg/ml				
	TQ	1, 3, 10, 100 µM/ml	Human granulocyte suspensions	LTC4 and LTB4 formation was inhibited. LTC4 synthase activity was significantly inhibited. LTA_4_ into LTC_4_ transformation in human blood cells was suppressed.	TQ effective concentration was close to TQ concentration used in animals.	(141)
*In vivo*	TQ	1 mg/kg	Allergic encephalomye litis rat model	Perivascular inflammation was reduced. Symptoms were reduced.	The anti-inflammatory effect was influenced by the increased glutathione level.	(142)
	TQ	15 mg/kg intraperitoneally (i.p.)	Multiple sclerosis mice model	Symptom developments were prevented in 90% of the subjects. Inflammation and symptoms were improved in 50% of the subjects.		(143)
	TQ	5 and 10 mg/kg	Acetic acid-induced colitis in rats	Myeloperoxidase activity, platelet activating factor, and histamine levels were restored. Glutathione levels were reduced.	Anti-inflammatory effect of TQ can be mediated by its antioxidant action.	(144)
	TQ	75 mg/kg of	Trinitrobenzene sulfonic acid (TNBS) induced colitis in rats	Proinflammatory cytokines were not changed. mRNA expression for IL-1β, IL-6, TNF-α, and IFN-γ in the colonic tissue was not affected. Histopathological changes were not reversed.	TQ anti-inflammatory effect in TNBS colitis was not proven.	(145)
	NSO	2.5 ml/kg, orally (p.o.)	TNBS induced colitis	LDH activity, TNF-α, IL-1β, IL-6 levels in blood were significantly decreased.	Anti-inflammatory and anti-oxidant activities were proven.	(146)
	NSO		TNBS induced colitis	TNF-α, IL-1β, and IL-6, lactate dehydrogenase, triglycerides, and cholesterol in serum were decreased. Neutrophil infiltration was inhibited.	Superoxide dismutase (SOD) activity was increased. Myeloperoxidase (MPO) levels were decreased.	(147)
	TQ	100 mg/kg	Chronic pancreatitis induced in rats	Amylase and lipase levels were changed. Il-1β and IL-18 levels were decreased.	MPO activity and the oxidative stress index (OSI) were decreased.	(148)
	NSO	0.66 ml and 1.55 ml/kg i.p.	Carrageenan-induced paw edema and cotton pellet granuloma formation in rats	Eicosanoids generation was inhibited.	Lipid peroxidation was inhibited.	(149)
	*Nigella sativa* seeds (NSS) essential oil	100, 200 and 400 µL/kg, p.o. or i.p.	Carrageenan-induced paw edema in rats	*N. sativa* essential oil had anti-inflammatory effect only in the i.p. administration.		(150)
	*N. sativa* aqueous extract	500 mg/kg	Carrageenan-induced paw edema	Inflammation was significantly reduced.	Results were comparable to aspirin (100 mg/kg).	(151)
	NSO	1g/ day	Rheumatoid arthritis patients	Anti-inflammatory IL-10 increased. Pro-inflammatory cytokines and TNF-α were non-significantly decreased.	Malondialdehyde (MDA) and NO serum levels were reduced.	(53)

↑: increase; ↓: decrease; Ca2+, calcium (II) ion; Na+, sodium ion; K+, potassium ion; Cl-, chloride ion; HMG-CoA reductase hydroxyl methyl glutaryl reductase, TC: total cholesterol; LDL: low-density lipoprotein cholesterol; TG: triglyceride; VLDL-C: very low-density lipoprotein cholesterol; HDL: high-density lipoprotein cholesterol

## Conclusion

Medicinal plants are used as alternative treatment therapies in several chronic diseases because of their reduced side effects and costs. Within this study, the potential anti-inflammatory and anti-oxidant effects of *N. sativa* in relation to different types of chronic conditions were investigated. Since inflammation and oxidative stress are strongly interconnected pathophysiological processes that are influencing themselves, the most recent references in regards to the experimental settings focused on both processes were analyzed. Studies have indicated that the use of black cumin in its different forms (as seeds, oil, or different extracts) can be used successfully in the treatment of various inflammatory diseases. Also, it was shown that because of its pharmacological properties like anti-inflammatory, anti-oxidant, and pro-apoptotic, *N. sativa* can be a valuable ally against cardiovascular diseases, diabetes and hematological malignancies. Future evidence based studies are needed to encourage the use of * N. sativa* in the management of these diseases or in the management of the side effects caused by the aggressive treatments.
